# Participatory Mathematical Modeling Approach for Policymaking during the First Year of the COVID-19 Crisis, Jordan

**DOI:** 10.3201/eid2909.221493

**Published:** 2023-09

**Authors:** Saverio Bellizzi, Nicholas Letchford, Keyrellous Adib, William J.M. Probert, Penelope Hancock, Lora Alsawalha, Alessio Santoro, Maria C. Profili, Ricardo Aguas, Christian Popescu, Lubna Al Ariqi, Lisa White, Wail Hayajneh, Nathir Obeidat, Pierre Nabeth

**Affiliations:** World Health Organization Jordan Country Office, Amman, Jordan (S. Bellizzi, L. Alsawalha, A. Santoro, M.C. Profili, C. Popescu);; World Health Organization Regional Office for Eastern Mediterranean, Cairo, Egypt (N. Letchford, K. Adib, W.J.M. Probert, Penelope Hancock, L. Al Ariqi, P. Nabeth);; Nuffield Department of Medicine, University of Oxford, Oxford, UK (R. Aguas, L. White);; SSM Health Cardinal Glennon Children's Hospital, St. Louis University, St. Louis, Missouri, USA (W. Hayajneh);; Jordan University Hospital, Amman (N. Obeidat)

**Keywords:** COVID-19, modeling, decision-making, curfew, coronavirus disease, SARS-CoV-2, severe acute respiratory syndrome coronavirus 2, viruses, respiratory infections, zoonoses, vaccine-preventable diseases, Jordan

## Abstract

We engaged in a participatory modeling approach with health sector stakeholders in Jordan to support government decision-making regarding implementing public health measures to mitigate COVID-19 disease burden. We considered the effect of 4 physical distancing strategies on reducing COVID-19 transmission and mortality in Jordan during March 2020–January 2021: no physical distancing; intermittent physical distancing where all but essential services are closed once a week; intermittent physical distancing where all but essential services are closed twice a week; and a permanent physical distancing intervention. Modeling showed that the fourth strategy would be most effective in reducing cases and deaths; however, this approach was only marginally beneficial to reducing COVID-19 disease compared with an intermittently enforced physical distancing intervention. Scenario-based model influenced policy-making and the evolution of the pandemic in Jordan confirmed the forecasting provided by the modeling exercise and helped confirm the effectiveness of the policy adopted by the government of Jordan.

Jordan reported ≈1.1 million confirmed COVID-19 cases and ≈12,500 deaths by the end of December 2021 (*1*), accounting for ≈6.0% of the total confirmed cases and ≈4.0% of the total number of deaths in the World Health Organization (WHO) Eastern Mediterranean Region (*1*). The COVID-19 epidemiologic curve in Jordan during the first 2 years of the pandemic followed distinct phases that reflected the complex interrelation between the natural evolution of the outbreak and the implementation of public health and social measures (PHSMs), which were also modulated in relation to the COVID-19 vaccination campaign (*2*) and the introduction of different variants of concern.

Jordan was particularly successful in flattening the epidemiologic curve during the first months of the pandemic until April 2020 because of implementation of strict PHSMs (*3*). However, the progressive easing of restrictions resulted in an exponential increase in cases, and the first 2 epidemic peaks in November 2020 and March 2021 led to ≈10,000 confirmed cases per day (*4*). Throughout that and subsequent phases of the pandemic, public health policies focused on reducing COVID-19 transmission and mortality in Jordan were supported by a participatory, epidemiologic scenario-based modeling approach. 

We provide an overview of lessons learned and challenges in conducting modeling efforts to simulate the transmission of SARS-CoV-2 in Jordan during the first year of the pandemic. Specifically, we assess the likely effectiveness of different combinations of physical distancing measures, and we describe the approach taken to ensure national level buy-in to the modeling results.

## Efficacy of Physical Distancing Interventions

During the earliest stages of the COVID-19 pandemic, in the absence of proven antiviral medication and vaccines, PHSMs represented the only option available for reducing COVID-19 community transmission and mortality (*5*). Among the wide variety of PHSMs applied in different settings, physical distancing interventions (PDIs) and curfews were considered among the most effective (*6*). For the purpose of our analysis, we considered PDIs to be interventions that require persons to maintain a physical distance of >1 m from other persons in all essential services (e.g., services conducted by grocery stores and healthcare facilities) and the closure of public places. The purpose of such interventions was ultimately to reduce the probability of COVID-19 transmission among persons (*7*). Evidence on the importance of this variety of PHSMs in limiting the transmission of COVID-19 emerged in Europe and Asia (*8*,*9*) and in the United States, where school closures have been found to reduce COVID-19 incidence and mortality rates by as much as 60% (*10*). Of note, several PHSMs, including PDIs, were substantially more effective when implemented while incidence rates remained low (*11*).

However, PDIs are unsustainable and may have wider-reaching detrimental effects. For example, home confinement considerably increased the rate of domestic violence in many countries, affecting women and children the most (*12*), and limited access to essential services for vulnerable populations (*13*–*17*). Therefore, tailored interventions that maintain persons’ livelihoods and keep economies functional while protecting persons at high risk need to be considered (*11*).

## Curfews and Physical Distancing Interventions in Jordan

**Figure 1:**
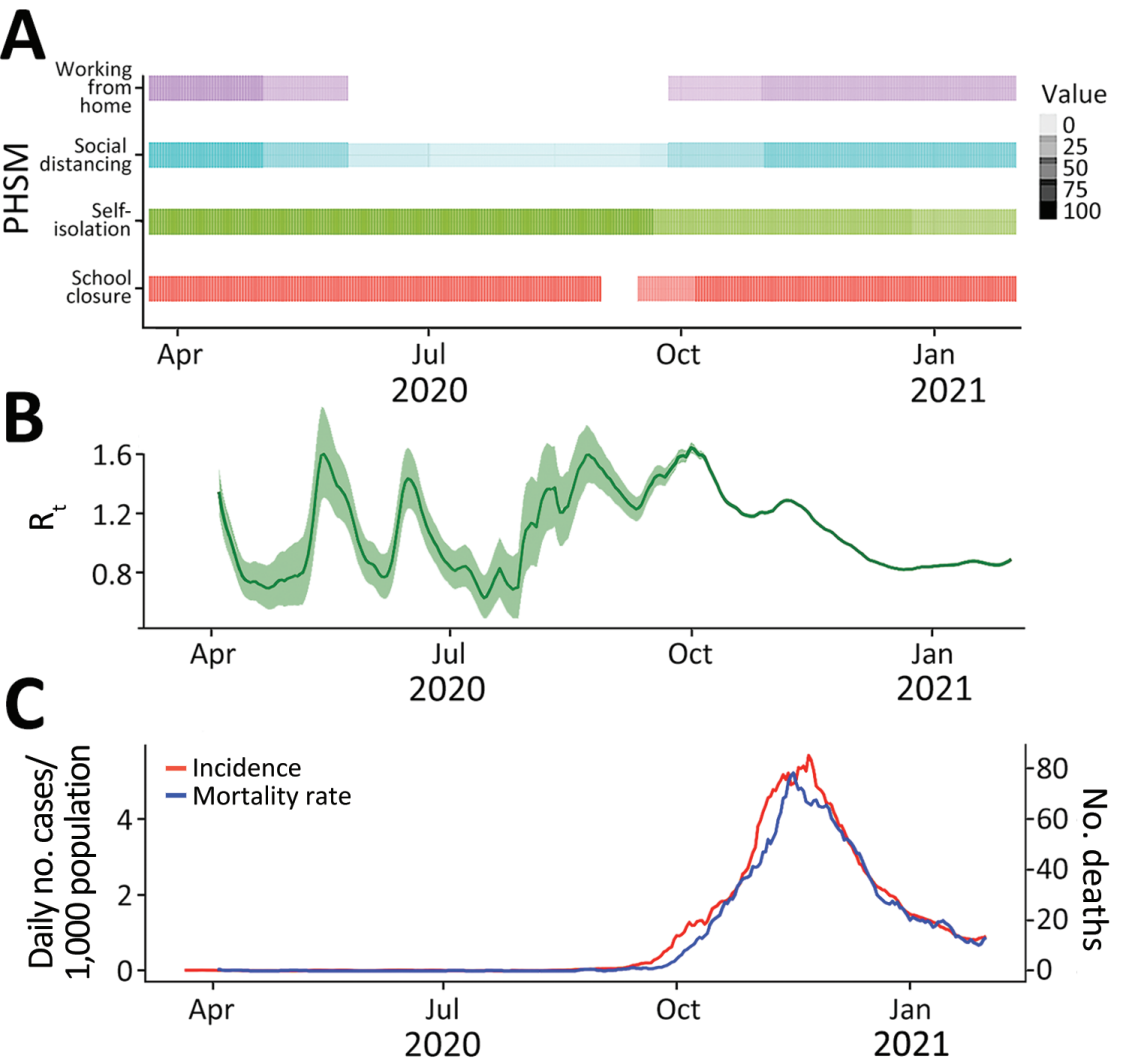
Epidemiologic indicators and PHSMs in a COVID-19 modeling study, Jordan, March 2020–January 2021. A) Timeline of implemented PHSMs. Colors indicate individual PHSMs; level of shading represents the coverage of each intervention in the timeline, ranging from 0% to 100%. B) Estimated R_t_, calculated using the EpiEstem package in R (https://CRAN.R-project.org/package=EpiEstim), which presents the number of new case-patients infected by an average case-patient at time t. Green shading indicates 95% CI. C) Daily incidence and mortality rates for COVID-19 in Jordan. PHSM, public health and social measure; Rt, effective reproduction number.

The PHSM strategy adopted in Jordan included imposing a nightly curfew (6 hours) from 12 AM to 6 AM, closing schools and universities, increasing community awareness of hygiene and enforcing a mask mandate in public places (*18*), and prohibiting mass gatherings (*19*). Community transmission in September 2020 triggered the imposition of an intermittent PDI, enforced on Fridays and Saturdays, lasting for 4 weeks. Shortly afterwards, physical distancing was only enforced on Fridays during October 2020–January 2021 (Figure 1). On those Fridays, all city activities, shops, and public places had to be closed (*19*). Furthermore, leaving the house was prohibited, except for persons who held a permit, such as healthcare personnel. Restrictions on other days of the week consisted of a 6-hour curfew period after midnight (from 12 AM to 6 AM), with no restriction on persons’ movement during the rest of the day (*19*). Such a unique approach was debated, and physical distancing for 1 day a week was questioned in terms of its healthcare benefit based on evidence (*20*).

The Jordan Ministry of Health, with the support of WHO, launched 3 rounds of a nationwide seroprevalence survey from the onset of the pandemic through the beginning of 2021. Findings revealed that seroprevalence steadily increased over time; only a tiny fraction of persons were seropositive in August 2020 (0.3%), a more than 20-fold increase was observed by October 2020 (7.0%), and up to one third of the overall population had been exposed by January 2021 (34.2%) (*4*).

## Using Mathematical Modeling in Decision-Making

In the context of infectious diseases, epidemiologic models play a critical role in anticipating the transmission of the disease and driving public health policies designed to limit illness and death (*21*). Specifically, epidemiologic models represent a tool for policy makers to design and evaluate targeted interventions. To do so, a range of factors specific to a setting are taken into consideration, such as demographic features, healthcare capacity, and the concurrent interaction among multiple PHSMs. When limited data are available, mathematical models can provide key elements to decision-makers on the effect of various future policy scenarios (*22*,*23*).

In Jordan, including relevant country stakeholders at each stage of the modeling process ensured that data were reliable and accurate and that the analysis was focused on addressing specific policy questions (*24*,*25*). The senior management of the Ministry of Health requested a series of scenarios on a regular basis (on average, once every 5–6 weeks) and worked directly with WHO to run the model and present the model’s findings to inform high-level and evidence-based decision-making. Starting after the second modeling round in October 2020, the Strategic Planning Department of the Jordanian Royal Hashemite Court supported those modeling techniques and bolstered them by expanding data availability, which was critical to initiate the process.

## Model Selection

At the onset of the pandemic, the WHO Jordan Country Office approached the Minister of Health to propose the use of mathematical modeling to estimate the epidemiologic outcomes under different scenarios. We selected and adapted the COVID-19 International Modeling Consortium (CoMo) model for implementing mathematical modeling analysis because of its suitability for conducting modeling analysis in low- to middle-income countries (*26*) and because it provided other desirable features, including the ongoing support from CoMo (*26*), an active team of software developers, and epidemiologic modelers. Additional resource requirements for implementing our participatory modeling approach were minimal (e.g., a stable internet connection, the R open-source statistical software [The R Foundation for Statistical Computing, https://www.r-project.org], and standard desktop applications).

The CoMo model is an age-dependent, deterministic, susceptible–exposed–infectious–recovered compartmental design that models transmission of SARS-CoV-2 in the population and can be used to estimate the relative effect of various PHSMs (Appendix). The model considers 5 levels of infection severity: asymptomatic, symptomatic, infections requiring hospitalization, infections requiring intensive care treatment, and infections requiring ventilated intensive care treatment. Infection severity and associated mortality rates are age-dependent, in that the proportion of infected persons requiring hospitalization and the proportion who die varies with age. In addition to predicting case and death rates at various timepoints, the CoMo model also incorporates 2 submodels: hospital and critical care requirements and implementation of public health and safety measures. The CoMo model incorporates a hospital submodel that suggests when hospital and critical care requirements will exceed the capacity of the country’s healthcare system (e.g., in terms of hospital beds, intensive care units, and ventilators available for use).

## Participatory Modeling of the COVID-19 Pandemic in Jordan

Participatory modeling approaches engage a range of stakeholders from academia, public health sectors, and government throughout the entire modeling process and promote the translation of model results into public health decision-making (*27*). We applied the participatory modeling process developed by WHO’s Eastern Mediterranean Region Office (EMRO) modeling support team to analyze the COVID-19 pandemic in Jordan. Specifically, WHO EMRO established a modeling support team in mid-March 2020 as part of the information management component within its COVID-19 Incident Management Support Team with the objective of addressing imminent decision-making needs and promoting awareness of how models work (*24*). When approaching the Minister of Health at the onset of the pandemic, the WHO Jordan Country Office proposed the use of the CoMo model. 

The participatory modeling began, therefore, with an initial meeting to communicate the modeling methodology and develop common expectations regarding the outcomes of the modeling exercise. The participants of this process included the WHO Jordan Country Office, the Minister of Health of Jordan, the Ministry of Health Secretary General for the COVID-19 portfolio (appointed to oversee COVID-19 response in Jordan), epidemiologic modeling researchers from the University of Oxford, and mathematical modelers, surveillance officers, and policy analysts from WHO EMRO. Although no specific declaration of interest was signed, there was no remuneration for any stakeholder.

We collected input parameters for the CoMo model by using a standardized template (developed in Excel [Microsoft, https://www.microsoft.com]) accompanied by a guidance document describing the model parameters and their definitions. We conducted 3 rounds of modeling analysis over a period of ≈3 months (November 2020–February 2021).

The participatory modeling process was instrumental in meeting recommended standards of practice associated with mathematical modeling for public health decision-making. Throughout the continued engagement of participants, communication of model uncertainty was reinforced, and key aspects of uncertainty, such as parameters related to viral transmission, were identified. Model outputs were routinely discussed among partners; satisfaction around model outputs paved the way for codevelopment of modeling results in the policy and decision-making process. In addition, patterns of reported and modeled COVID-19 disease and mortality were used for discussions regarding public health surveillance to identify possible challenges and misreporting of COVID-19 with specialists at the Ministry of Health, concerns that were evident from the experience of COVID-19 collaborative modeling in the Philippines by the WHO Western Pacific Region Office (*28*).

The participatory process helped to define the context for the modeling exercise, including questions of importance to policymakers, and make it easier to collect country-specific model inputs (Appendix). Those communications also were productive in developing interpretations of the analysis that were relevant and useful to all participants.

## Scenario-Based Modeling of the COVID-19 Pandemic in Jordan

We considered 4 scenarios in the analysis: the baseline scenario and 3 other scenarios (A, B, and C). All scenarios considered interventions that were designed to reduce the rate at which persons come into contact with each other, stemming COVID-19 transmission in Jordan. Common to each scenario are 2 parameters that can be used to define the extent of the PDI: coverage and adherence. Coverage refers to the percentage of the population that is following physical distancing regulations; adherence refers to the extent individual persons follow those guidelines. An intervention with low adherence but high coverage would mean that most of the population loosely follow the physical distancing regulations. Conversely, an intervention with high adherence but low coverage would mean a small percentage of the population follow the physical distancing regulations to a high standard. All other parameters in the model were held constant throughout the duration of the simulation. We developed the scenarios considered through an iterative process of engaging with relevant policy makers, updating the scenarios as more information became available (since the last analysis), and adapting the scenarios to reflect the effect of potential future changes to PHSMs.

The baseline scenario considers the situation of no government intervention but assumed 50% of the population would continue to physically distance themselves. This percentage was suggested by public health experts in Jordan and is in line with available literature (*29*). Scenario A assumed the Jordan population would physically distance themselves for a period of 24 hours every Friday (considering Friday prayer observance), applying to all but basic services, such as hospitals and grocery stores. No government restrictions were assumed to be imposed on the other days of the week, yet, as in the baseline scenario, we assumed a portion of the population (50%) would continue to practice a degree of physical distancing regardless of government guidelines. Similarly, scenario B is an extension of scenario A in that all but essential services were required to close over the entire weekend, reducing contacts as much as possible. Last, scenario C, being the most extreme scenario considered in our analysis, assumed all but essential services were closed for the entire week until the end of the simulation period. Consistent across each scenario we assumed the interventions came into effect on October 31, 2020, and lasted until the end of the simulation period on January 31, 2021.

## Estimated Effect of Continuation of Planned Measures on Health Outcomes

**Figure 2:**
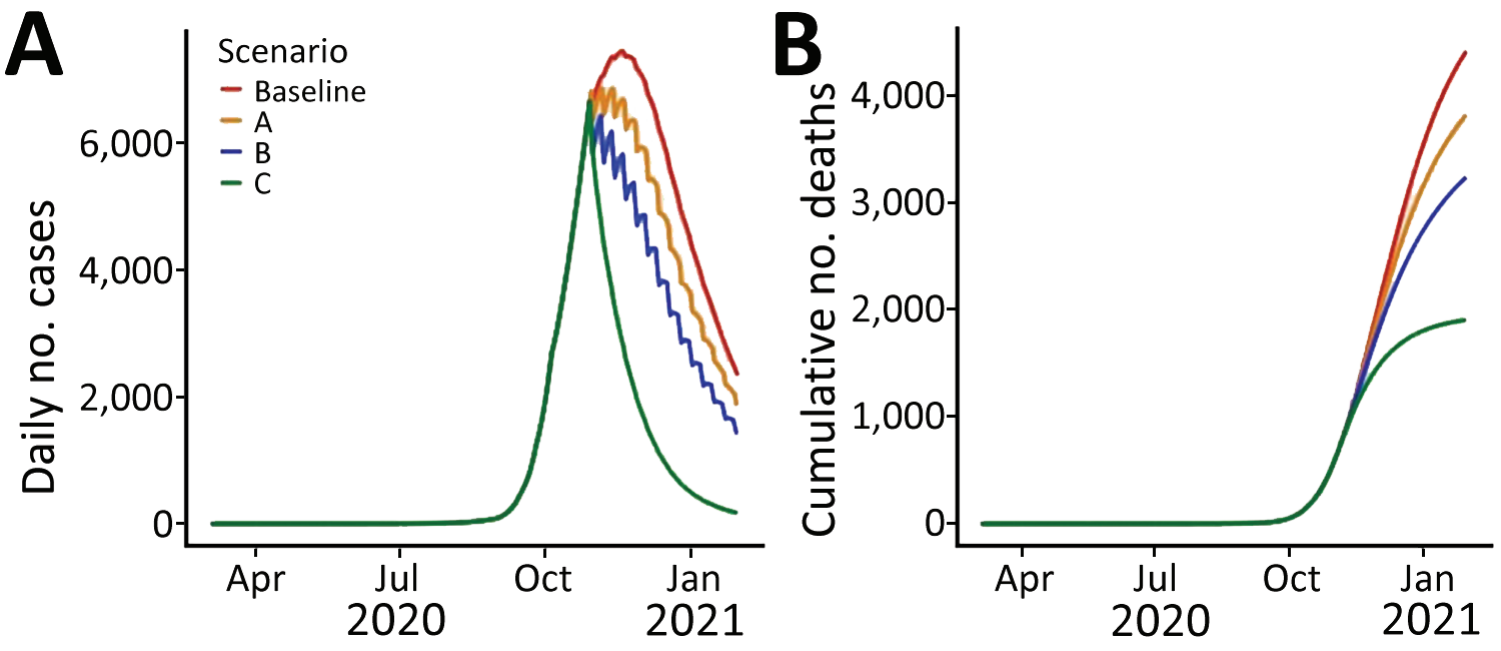
Model-predicted reported number of COVID-19 cases and cumulative number of associated deaths under scenarios A, B, C, and the baseline scenario in a COVID-19 modeling study, Jordan, March 2020–January 2021. Scenario A assumes the entire population, excepting essential services, will physically distance themselves for 24 hours every Friday while reverting to their usual behavior on the other days of the week. Scenario B assumes the population will physically distance themselves for the entire weekend (Friday and Saturday) while reverting to their usual behavior throughout the week. Scenario C assumes the entire population, except for essential services, will physically distance themselves for the entire week while never reverting to their usual behavior. Baseline scenario assumes no government intervention and half the population instinctively physically distancing themselves to avoid infection. Common to each scenario are 2 parameters used to define the extent of the physical distancing intervention: coverage, which refers to the percentage of the population following physical distancing regulations, and adherence, which refers to the extent to which individual persons follow those guidelines. On days when the interventions are not enforced, simulations assume 80% adherence and 50% coverage of the population practice physical distancing, while on days when the interventions are enforced it is assumed that 80% adherence and 90% coverage of the population physically distance themselves.

The timing of the predicted peak incidence, which was estimated to occur in mid-November 2020, varied only marginally across the different scenarios (Figure 2, panel A). However, soon after the interventions in scenarios A, B, and C were implemented, their effect was observed in reduced incidence (Figure 2, panel A) and cumulative mortality (Figure 2, panel B). Unsurprisingly, the most impactful scenario was scenario C, where a sharp and rapid reduction in cases and deaths was predicted to occur shortly after implementation. However, the economic cost of such an intervention would likely have been substantial for the population.

## Exploring Variation in Efficacy of Different Scenarios

**Figure 3:**
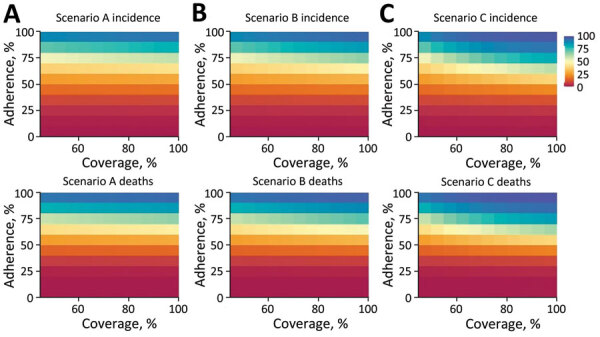
Model-predicted heat map showing percentage reduction in COVID-19 incidence (top row) and deaths (bottom row) in a COVID-19 modeling study in Jordan under 3 different scenarios (A, B, and C), relative to the baseline scenario, aggregated for the period November 2020–January 31, 2021. Dark blue corresponds to nearly 100% reduction in incidence and cases relative to the baseline scenario; dark red corresponds to 0% reduction. Scenario A assumes the entire population, excepting essential services, will physically distance themselves for 24 hours every Friday while reverting to their usual behavior on the other days of the week. Scenario B assumes the population will physically distance themselves for the entire weekend (Friday and Saturday) while reverting to their usual behavior throughout the week. Scenario C assumes the entire population, except for essential services, will physically distance themselves for the entire week while never reverting to their usual behavior. Baseline scenario assumes no government intervention and half the population instinctively physically distances themselves to avoid infection. Common to each scenario are 2 parameters used to define the extent of the physical distancing intervention: coverage, which refers to the percentage of the population following physical distancing regulations, and adherence, which refers to the extent to which individual persons follow those guidelines. The coverage parameter was varied between values of 50% and 100% (presented on the horizontal axis of each heat map) on the days when the physical distancing intervention was enforced. On respective days when the interventions were not enforced, simulations assume the coverage was constant at 50%. The adherence parameter varied between 0% and 100% (presented on the vertical axis of each heat map), remaining constant throughout each simulation.

We estimated the effect of scenarios A, B, and C in terms of the percentage reduction of COVID-19 cases and deaths during November 2020–January 2021 relative to the baseline scenario (Figure 3). The coverage of the PDI in each scenario was assumed to only be relevant during the days of the week the intervention was enforced. During the nonintervention days of the week, we assumed 50% of the population continued to practice physical distancing regardless of government guidelines. Consistently across each scenario, the model estimated that the greatest reduction in COVID-19 incidence and death was associated with increasing adherence to the respective physical distancing guidelines implemented by the government. When the adherence of the population was low, increasing the coverage of the PDI had relatively little effect on reducing disease. Conversely, however, if the adherence of persons who follow government regulations was high (>80%), the model estimated that increasing the coverage of the population had compounded effects on reducing COVID-19 disease incidence and death.

The greatest effect was observed under scenario C, with high coverage and high adherence (97% reduction in cases and deaths relative to the baseline scenario, assuming 100% coverage and adherence). However, assuming adherence and coverage >90% for either scenario A or B, the model predicted that reported cases and deaths would have reduced by ≈90% relative to the baseline scenario. In contrast, any scenario (either A, B, or C) with low coverage (<25%) had almost no effect, decreasing disease incidence and death by as little as 10% relative to the baseline scenario. The difference in disease incidence and death between scenarios A and C equates to roughly 7% fewer cases and deaths (assuming the coverage and adherence are both high [>90%]). As coverage and particularly adherence decreases, diseases incidence and death increase rapidly. Those results suggest that implementing scenario C during October 31, 2020–January 31, 2021, would be only marginally beneficial at reducing COVID-19 disease and death compared with scenario A or B with high coverage and adherence. The findings of our analysis and the subsequent decision-making was supported by epidemiologic and economic modeling for COVID-19 policy in Australia; although tighter stringency PHSMs remarkably reduced cumulative infections in that country, that effect had the tradeoff of higher expected societal economic losses (*29*). Therefore, ranking of policy options should be based on optimality and cost-effectiveness, possibly leading to a mix of higher-stringency PHSMs (*30*).

**Figure 4:**
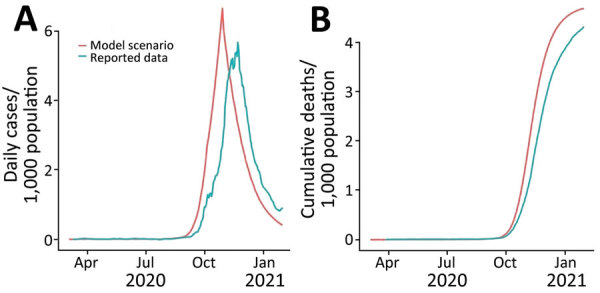
Comparison of COVID-19 incidence (A) and cumulative deaths daily (B) under model scenario A compared with reported data in a COVID-19 modeling study, Jordan, March 2020–January 2021. Scenario A assumes the entire population, excepting essential services, will physically distance themselves for 24 hours every Friday while reverting to their usual behavior on the other days of the week. The scenario is defined by 2 key parameters: coverage and adherence. On days when the physical distancing intervention was enforced, the simulation assumes 60% of the population is following physical distancing regulations (coverage) and that those persons spend 80% of their time adhering to the intervention (adherence).

**Figure 5:**
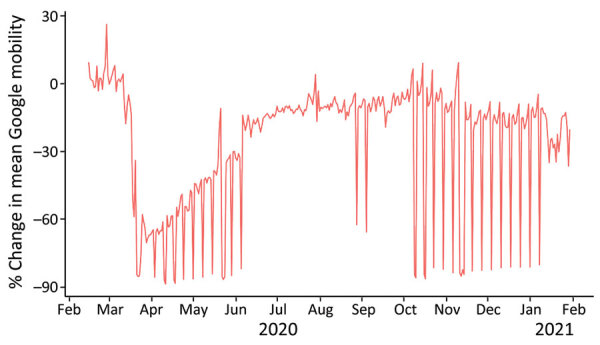
Percentage changes in mean mobility among the population, Jordan, February 2020–January 2021, including around retail and recreational facilities, grocery and pharmacy stores, parks, and transit locations. Google mobility data are used as a proxy for the population’s coverage and adherence to COVID-19–related physical distancing interventions.

We retrospectively compared the results of scenario A to historical reported data (Figure 4). We found the incidence under scenario A closely resembled the reported data for an assumed coverage of 60% and adherence of 80% and even more so for cumulative mortality (Figure 4). The coverage and adherence parameters for another scenario (Figure 5) closely resemble the reported Google mobility data for Jordan (*31*). We considered the average of the Google mobility data reported from retail and recreational facilities, grocery and pharmacy stores, and parks and transit locations. Changes in the average Google mobility data occurred on weekly intervals, representing the reduced mobility of persons during the weekend (Figure 5).

## Challenges and Limitations

As in all modeling studies, we made various assumptions in this analysis. We cannot accurately estimate COVID-19 transmission rates and the effective reproduction number (R_t_) when the burden of COVID-19 in the country is underestimated because of underreporting of cases and associated deaths. This limitation prevented us from performing model fitting, for example, using Bayesian particle filtering methods, to estimate the actual dynamics of COVID-19 and perform inference on key parameters such as the basic reproduction number (R_0_). Moreover, although our models included age-specific mixing patterns, geographic location–specific mixing patterns were ignored. This analysis modeled Jordan as a whole, whereas differences between governorates may have warranted a spatially explicit approach to modeling. The analysis did not account for the introduction of variants of concern and assumed that natural infection provided lifelong protection against reinfection. Ensuring policy makers understand the limitations of these assumptions through clear communication is vital to ensure the model’s relevance.

## Conclusions

COVID-19 modeling has been a substantial achievement (*32*). Strong and consistent national support and inputs from a wide range of critical stakeholders, such as the Ministry of Health and the Royal Hashemite Court, ensured that estimations of relative effect have been constantly refined over time.

The participatory scenario-based approach we describe considered the effect of intermittent PDIs on reducing COVID-19 transmission in Jordan. We show that enforcing a PDI with no intermittent periods is only marginally beneficial to reducing COVID-19 disease burden compared with an intermittently enforced PDI. The evolution of the pandemic in Jordan confirmed the forecasting provided by the modeling exercise and helped confirm the effectiveness of the policy adopted by the government of Jordan. The insights from scenario-based modeling influenced the implementation of PHSMs and PDIs; specifically, scenario-based models were used to updating PHSM and PDI guidelines in addition to other evidence-based actions, such as infection prevention and control (*33*).

By interacting directly with the policy decision-makers, we were able to define the context of the modeling exercise and address specific policy questions they posed. Furthermore, communicating what mathematical modeling is capable of and its limitations at every stage of the analysis was vital to the success of the project. This level of engagement strengthened communication between stakeholders and encouraged insights learned through the modeling process to be incorporated into policy decisions.

This modeling initiative for the pandemic confirmed the comparative advantage in providing hands-on support to national health authorities for developing evidence-based policies. The participatory approach in running COVID-19 modeling research provided the chance to convey the model’s caveats and limitations and disseminate modeling results among governing bodies and partners as appropriate. By leveraging and investing in WHO resources and providing essential assistance for the pandemic (e.g., procurement, research, and capacity building), WHO created crucial evidence to help with decision-making within and beyond Jordan’s health sector.

Appendix Additional information about participatory mathematical modelling approach for policymaking during the first year of the COVID-19 crisis, Jordan.

## References

[1] World Health Organization. Weekly epidemiological update on COVID-19—28 December 2021 [cited 2023 Apr 1]. https://www.who.int/publications/m/item/weekly-epidemiological-update-on-covid-19—28-december-2021

[2] Bellizzi S, Aidyralieva C, Alsawhala L, Al-Shaikh A, Santoro A, Profili MC. Vaccination for SARS-CoV-2 of migrants and refugees, Jordan. Bull World Health Organ. 2021;99:611. 10.2471/BLT.21.285591 34475595 PMC8381092

[3] Brookings Institution. Policy and institutional responses to COVID-19 in the Middle East and North Africa: Jordan [cited 2023 Apr 1]. https://www.brookings.edu/research/policy-and-institutional-responses-to-covid-19-in-the-middle-east-and-north-africa-jordan

[4] Bellizzi S, Alsawalha L, Sheikh Ali S, Sharkas G, Muthu N, Ghazo M, et al. A three-phase population based sero-epidemiological study: Assessing the trend in prevalence of SARS-CoV-2 during COVID-19 pandemic in Jordan. One Health. 2021;13:100292. 10.1016/j.onehlt.2021.100292 34295958 PMC8272624

[5] Qualls N, Levitt A, Kanade N, Wright-Jegede N, Dopson S, Biggerstaff M, et al.; CDC Community Mitigation Guidelines Work Group. Community mitigation guidelines to prevent pandemic influenza—United States, 2017. MMWR Recomm Rep. 2017;66:1–34. 10.15585/mmwr.rr6601a1 28426646 PMC5837128

[6] Haug N, Geyrhofer L, Londei A, Dervic E, Desvars-Larrive A, Loreto V, et al. Ranking the effectiveness of worldwide COVID-19 government interventions. Nat Hum Behav. 2020;4:1303–12. 10.1038/s41562-020-01009-0 33199859

[7] World Health Organization. COVID-19: physical distancing [cited 2023 Apr 1]. https://www.who.int/westernpacific/emergencies/covid-19/information/physical-distancing

[8] Flaxman S, Mishra S, Gandy A, Unwin HJT, Mellan TA, Coupland H, et al.; Imperial College COVID-19 Response Team. Estimating the effects of non-pharmaceutical interventions on COVID-19 in Europe. Nature. 2020;584:257–61. 10.1038/s41586-020-2405-7 32512579

[9] Park YJ, Choe YJ, Park O, Park SY, Kim YM, Kim J, et al.; COVID-19 National Emergency Response Center, Epidemiology and Case Management Team. Contact tracing during coronavirus disease outbreak, South Korea, 2020. Emerg Infect Dis. 2020;26:2465–8. 10.3201/eid2610.201315 32673193 PMC7510731

[10] Auger KA, Shah SS, Richardson T, Hartley D, Hall M, Warniment A, et al. Association between statewide school closure and COVID-19 incidence and mortality in the US. JAMA. 2020;324:859–70. 10.1001/jama.2020.14348 32745200 PMC7391181

[11] Talic S, Shah S, Wild H, Gasevic D, Maharaj A, Ademi Z, et al. Effectiveness of public health measures in reducing the incidence of covid-19, SARS-CoV-2 transmission, and covid-19 mortality: systematic review and meta-analysis. BMJ. 2021;375:e068302. 10.1136/bmj-2021-068302 34789505 PMC9423125

[12] Bellizzi S, Nivoli A, Lorettu L, Farina G, Ramses M, Ronzoni AR. Violence against women in Italy during the COVID-19 pandemic. Int J Gynaecol Obstet. 2020;150:258–9. 10.1002/ijgo.13270 32533860 PMC9087562

[13] Bellizzi S, Nivoli A, Lorettu L, Ronzoni AR. Human rights during the COVID-19 pandemic: the issue of female genital mutilations. Public Health. 2020;185:53–4. 10.1016/j.puhe.2020.05.037 32563099 PMC7247461

[14] Bellizzi S, Pichierri G, Napodano CMP, Picchi S, Fiorletta S, Panunzi MG, et al. Access to modern methods of contraception in Italy: Will the COVID-19 pandemic be aggravating the issue? J Glob Health. 2020;10:020320. 10.7189/jogh.10.020320 33110522 PMC7561212

[15] Bellizzi S, Alsawalha L, Samawi L, Al-Shaikh A, Alfar H, Muthu N, et al. The impact of the SARS-CoV-2 pandemic on mental health in vulnerable population settings: the case of Jordan. Front Psychiatry. 2021;12:692541. 10.3389/fpsyt.2021.692541 34366920 PMC8339266

[16] Bellizzi S, Ronzoni AR, Pichierri G, Cegolon L, Salaris P, Panu Napodano CM, et al. Safe abortion amid the COVID-19 pandemic: The case of Italy. Int J Gynaecol Obstet. 2020;150:254–5. 10.1002/ijgo.13233 32437584 PMC9087612

[17] Bellizzi S, Muthu N, Khader Y, Boukerdenna H, Darwish D, Al-Sheikh A, et al. COVID-19 and non-communicable diseases in complex vulnerable populations: evidence from Jordan. Prim Health Care Res Dev. 2023;24:e8. 10.1017/S1463423622000731 36661207 PMC9884531

[18] Bubbico L, Mastrangelo G, Larese-Filon F, Basso P, Rigoli R, Maurelli M, et al. Community use of face masks against the spread of COVID-19. Int J Environ Res Public Health. 2021;18:3214. 10.3390/ijerph18063214 33808861 PMC8003592

[19] AlRyalat SA, Elubous KA, Al-Ebous AD, Mahafzah A. Impact of a single-day lockdown on COVID-19: an interrupted time series analysis. Cureus. 2021;13:e17299. 10.7759/cureus.17299 34552834 PMC8449516

[20] Al Mostafa M, Abu Ruman YF, Hashim HT, Ramadhan MA. Friday lockdown in Jordan: Good lessons to be learned from Jordan? [letter]. Ethics Med Public Health. 2021;18:100663. 10.1016/j.jemep.2021.100663

[21] Aylett-Bullock J, Gilman RT, Hall I, Kennedy D, Evers ES, Katta A, et al. Epidemiological modelling in refugee and internally displaced people settlements: challenges and ways forward. BMJ Glob Health. 2022;7:e007822. 10.1136/bmjgh-2021-007822 35264317 PMC8915287

[22] Teslya A, Pham TM, Godijk NG, Kretzschmar ME, Bootsma MCJ, Rozhnova G. Impact of self-imposed prevention measures and short-term government-imposed social distancing on mitigating and delaying a COVID-19 epidemic: A modelling study. PLoS Med. 2020;17:e1003166. 10.1371/journal.pmed.1003166 32692736 PMC7373263

[23] McBryde ES, Meehan MT, Adegboye OA, Adekunle AI, Caldwell JM, Pak A, et al. Role of modelling in COVID-19 policy development. Paediatr Respir Rev. 2020;35:57–60. 32690354 10.1016/j.prrv.2020.06.013 PMC7301791

[24] Adib K, Hancock PA, Rahimli A, Mugisa B, Abdulrazeq F, Aguas R, et al. A participatory modelling approach for investigating the spread of COVID-19 in countries of the Eastern Mediterranean Region to support public health decision-making. BMJ Glob Health. 2021;6:e005207. 10.1136/bmjgh-2021-005207 33762253 PMC7992384

[25] Aguas R, White L, Hupert N, Shretta R, Pan-Ngum W, Celhay O, et al.; CoMo Consortium. Modelling the COVID-19 pandemic in context: an international participatory approach. BMJ Glob Health. 2020;5:e003126. 10.1136/bmjgh-2020-003126 33361188 PMC7759758

[26] CoMo Consortium; The COVID-19 International Modelling Consortium. [cited 2023 Apr 1]. https://como.bmj.com

[27] Adams S, Rhodes T, Lancaster K. New directions for participatory modelling in health: Redistributing expertise in relation to localised matters of concern. Glob Public Health. 2022;17:1827–41. 10.1080/17441692.2021.1998575 34775919

[28] Hughes A, Ragonnet R, Jayasundara P, Ngo HA, de Lara-Tuprio E, Estuar MRJ, et al. COVID-19 collaborative modelling for policy response in the Philippines, Malaysia and Vietnam. Lancet Reg Health West Pac. 2022;29:100563. 10.1016/j.lanwpc.2022.100563 35974800 PMC9371475

[29] Kim HY, Bershteyn A, McGillen JB, Shaff J, Sisti J, Ko C, et al. Social distancing and mask-wearing could avoid recurrent stay-at-home restrictions during COVID-19 respiratory pandemic in New York City. Sci Rep. 2022;12:10312. 10.1038/s41598-022-13310-1 35725991 PMC9207433

[30] Szanyi J, Wilson T, Howe S, Zeng J, Andrabi H, Rossiter S, et al. Epidemiologic and economic modelling of optimal COVID-19 policy: public health and social measures, masks and vaccines in Victoria, Australia. Lancet Reg Health West Pac. 2023;32:100675. 10.1016/j.lanwpc.2022.100675 36694478 PMC9851841

[31] Google. COVID-19 community mobility reports [cited 2023 Apr 1]. https://www.google.com/covid19/mobility

[32] World Health Organization. WHO supports generating evidence for decision-making in Jordan during COVID-19 [cited 2023 Apr 1]. https://www.who.int/about/accountability/results/who-results-report-2020-mtr/country-story/2020/jordan

[33] Tarif AB, Ramadan M, Yin M, Sharkas G, Ali SS, Gazo M, et al. Infection prevention and control risk factors in health workers infected with SARS-CoV-2 in Jordan: A case control study. PLoS One. 2022;17:e0271133. 10.1371/journal.pone.0271133 35802587 PMC9269456

