# Supplementary material for: Participatory Mathematical Modeling Approach for Policymaking during the First Year of the COVID-19 Crisis, Jordan
Source: Emerg Infect Dis. 2023 Sep;29(9):1738–46. doi: 10.3201/eid2909.221493 (PMC10461658; doi:10.3201/eid2909.221493)
Supplement: Appendix — Additional information about participatory mathematical modelling approach for policymaking during the first year of the COVID-19 crisis, Jordan. [file 22-1493-Techapp-s1.pdf]

Article DOI: https://doi.org/10.3201/eid2909.221493

EID cannot ensure accessibility for supplementary materials supplied by authors. Readers who have difficulty accessing supplementary content should contact the authors for assistance.

Participatory Mathematical Modeling Approach for Policymaking during the First Year of the COVID-19 Crisis, Jordan

Appendix

Model Description

Our participatory modeling approach used the COVID-19 International Modeling Consortium (CoMo) model (V17.20) (*1*). The model has been actively developed throughout the COVID19 pandemic by a global consortium of scientists including members of the EMRO modeling support team. The code is open source and publicly available. The version of the model used in this analysis (version 17.2) is available at https://github.com/ocelhay/como/releases/tag/v17.2.0.

The CoMo model is an age-dependent, deterministic, SEIR (Susceptible - Exposed - Infectious – Recovered) compartmental model that models transmission of SARS-CoV-2 in the population and can be used to investigate the relative impact of a variety of PHSM. The model considers five levels of infection severity: asymptomatic, symptomatic, infections requiring hospitalization, intensive care treatment, and ventilated intensive care treatment. Infection severity and associated mortality are age-dependent, in that the proportion of infected individuals requiring hospitalization, and the proportion that die, varies with age. In addition to predicting case and death rates at various time points, the CoMo model also incorporates two sub-models: hospital and critical care requirements and implementation of public health and safety measures. The CoMo model incorporates a hospital sub-model that suggests when hospital and critical care requirements will exceed the capacity of the country’s healthcare system, including treatment in hospital beds, ICUs, and ventilators.

The CoMo model also incorporates an explicit representation of various PHSM to mitigate the spread of SARS CoV-2. These measures are: self-isolation of symptomatic individuals and self-quarantine of members of their household, screening of the contacts of individuals with a positive diagnostic test result, mass testing, school closure, workplace closure, physical distancing measures, border closure, shielding elderly individuals, handwashing, and mask-wearing. For each PHSM, users can vary the timing and duration, as well as the coverage, defined as the proportion of the population that adheres to the intervention, and adherence, defined as the proportion of time in a given day that an average individual adheres to the intervention. The coverage and adherence values act on the age- and location-dependent contact matrices (*2*). Coverage values of each PHSM in the model may vary over time to simulate interventions being relaxed and reinstated according to policy mandates.

Model Equations

This Supplemental Material provides an outline of the equations and parameters describing the CoMo model (*1*). The CoMo model is written in R and deployed via a Shiny App (comomodel.net). Equations in this document are based upon the solver in v16.6.0 of the comoOdeCpp R package, which is paired with v17.2 of the CoMo model. This combination of model and solver were used in the analyses. An R script of the solver (for use with the deSolve package) is available in the tests folder of the comoOdeCpp package.

Notation in this document aims to stay consistent with 1) the code in the solver, 2) the model equations in the original paper of Aguas et al. (*1*) (which were not published for v17.2 of the model), 3) the code in R for use with the deSolve package. A few adjustments have been made for consistency across the notation (e.g., durations all use the same symbol).

Differential equations

For clarity and consistency, derivatives have been written in the following form:

derivative = ...

transmission terms

+ disease processes

+ quarantine

+ aging

+ natural mortality

+ births

Susceptible individuals

$$\frac{dS}{dt} = -S\lambda - vAS + \omega R + v_d V - q_r S + 1/v_q QS + A_{age} S - \mu S + b$$

Susceptible individuals, currently quarantining

$$\frac{dQS}{dt} = -\lambda_q QS + q_r S - 1/v_q QS + A_{age} QS - \mu QS$$

Infected and incubating

$$\frac{dE}{dt} = S\lambda - \gamma E - vAE - q_r E + 1/v_q QE + A_{age} E - \mu E$$

Infected and incubating, currently quarantining

$$\frac{dQE}{dt} = \lambda_q QS - \gamma QE + q_r E - 1/v_q QE + A_{age} QE - \mu QE$$

Infectious symptomatic individuals

$$\frac{dI}{dt} = \ldots$$

$$\gamma(1 - p_{clin})(1 - s_{screen})(1 - p_{ihr})(1 - A_u u_E)E \ldots$$

$$+\gamma(1 - p_{clinV})(1 - s_{screen})(1 - \sigma_{EV} p_{ihr})(1 - A_u u_{EV})EV \ldots$$

$$+\gamma(1 - p_{clinVR})(1 - s_{screen})(1 - \sigma_{EVR} p_{ihr})(1 - A_u u_{EVR})EVR \ldots$$

$$+\gamma(1 - p_{clinR})(1 - s_{screen})(1 - \sigma_{ER} p_{ihr})(1 - A_u u_{ER})ER \ldots$$

$$-vA_v I - v_i I - u_I A_u I \ldots$$

$$-q_r I + 1/v_q QI + A_{age} I - \mu I$$

Infectious individuals (currently quarantining)

$$\frac{dQI}{dt} = \gamma(1 - p_{ihr})(1 - p_{clin})QE \ldots$$

$$+\gamma(1 - \sigma_{EV} p_{ihr})(1 - p_{clinV})QEV \ldots$$

$$+\gamma(1-\sigma_{EVR}p_{ihr})(1-p_{clinVR})QEVR...$$

$$+\gamma(1-\sigma_{ER}p_{ihr})(1-p_{clinR})QER...$$

$$-\nu_i QI + q_r I - 1/\nu_q QI + A_{age}QI - \mu QI$$

Symptomatic and reported cases

$$\frac{dCL}{dt} = \gamma p_{clin}(1-A_u u_E)(1-c_{iso})(1-p_{ihr})(1-q_r)E...$$

$$+\gamma p_{clinV}(1-A_u u_{EV})(1-c_{iso})(1-\sigma_{EV}p_{ihr})(1-q_r)EV...$$

$$+\gamma p_{clinVR}(1-A_u u_{EVR})(1-c_{iso})(1-\sigma_{EVR}p_{ihr})(1-q_r)EVR...$$

$$+\gamma p_{clinR}(1-A_u u_{ER})(1-c_{iso})(1-\sigma_{ER}p_{ihr})(1-q_r)ER...$$

$$-u_{CL}A_u CL - \nu_i CL + 1/\nu_q QC + A_{age}CL - \mu CL$$

$$\frac{dQC}{dt} = ...$$

$$\gamma p_{clin}(1-c_{iso})(1-A_u u_E)(1-p_{ihr})q_r E...$$

$$+\gamma p_{clinV}(1-c_{iso})(1-A_u u_{EV})(1-\sigma_{EV}p_{ihr})q_r EV...$$

$$+\gamma p_{clinVR}(1-c_{iso})(1-A_u u_{EVR})(1-\sigma_{EVR}p_{ihr})q_r EVR...$$

$$+\gamma p_{clinR}(1-c_{iso})(1-A_u u_{ER})(1-\sigma_{ER}p_{ihr})q_r ER...$$

$$+\gamma(1-p_{ihr})p_{clin}QE...$$

$$+\gamma(1-\sigma_{EV}p_{ihr})p_{clinV}QEV...$$

$$+\gamma(1-\sigma_{EVR}p_{ihr})p_{clinVR}QEVR...$$

$$+\gamma(1-\sigma_{ER}p_{ihr})p_{clinR}QER...$$

$$-\nu_i QC - 1/\nu_q QC + A_{age}QC - \mu QC$$

Self-isolating

$$\frac{dX}{dt} = ...$$

$$\gamma c_{iso}(1-A_u u_E)p_{clin}(1-p_{ihr})E...$$

$$+\gamma s_{screen}(1-A_u u_E)(1-p_{clin})(1-p_{ihr}\quad)E\ldots$$

$$+\gamma c_{iso}(1-A_u u_{EV})p_{clinV}(1-p_{ihr}\quad)EV\ldots$$

$$+\gamma s_{screen}(1-A_u u_{EV})(1-p_{clinV})(1-p_{ihr}\quad)EV\ldots$$

$$+\gamma c_{iso}(1-A_u u_{EVR})p_{clinVR}(1-p_{ihr}\quad)EVR\ldots$$

$$+\gamma s_{screen}(1-A_u u_{EVR})(1-p_{clinVR})(1-p_{ihr}\quad)EVR\ldots$$

$$+\gamma c_{iso}(1-A_u u_{ER})p_{clinR}(1-p_{ihr}\quad)ER\ldots$$

$$+\gamma s_{screen}(1-A_u u_{ER})(1-p_{clinR})(1-p_{ihr}\quad)ER\ldots$$

$$-\nu_i X + A_{age}X - \mu X$$

Quarantined due to testing

$$\frac{dZ}{dt}=\ldots$$

$$\gamma u_E A_u(1-p_{ihr})E\ldots$$

$$+\gamma u_{EV} A_u(1-p_{ihr})EV\ldots$$

$$+\gamma u_{EVR} A_u(1-p_{ihr})EVR\ldots$$

$$+\gamma u_{ER} A_u(1-p_{ihr})ER\ldots$$

$$+u_I A_u I + u_{CL} A_u CL + u_{HC} A_u HC + u_{HCICU} A_u HCICU + u_{HCV} A_u HCV\ldots$$

$$-1/\nu_{iso}Z - \mu Z$$

Recovered and immune individuals

$$\frac{dR}{dt}= -\lambda\sigma_R R + \nu_i I \ + \ \nu_i X + \nu_i CL - \omega R + \ldots$$

$$\nu_H p_{O2}\,(1-d_{O2}\delta_{HO2})p_{ifr}H+\ldots$$

$$\nu_H(1-p_{O2})\,(1-\delta_H)p_{ifr}H+\ldots$$

$$\nu_{HC} p_{O2}\,(1-\delta_{HCO2})p_{ifr}HC+\ldots$$

$$\nu_{HC}(1-p_{O2})\,(1-\delta_{HC})p_{ifr}HC+\ldots$$

$$\nu_{ICU}p_{O2}\ (1 - d_{O2}\delta_{ICUO2})p_{ifr}ICU + \ldots$$

$$\nu_{ICU}(1 - p_{O2})\ (1 - \delta_{ICU})p_{ifr}ICU + \ldots$$

$$\nu_{ICUC}p_{O2}\ (1 - d_{O2C}\delta_{ICUCO2})p_{ifr}ICUC + \ldots$$

$$\nu_{ICUC}(1 - p_{O2})\ (1 - \delta_{ICUC})p_{ifr}ICUC + \ldots$$

$$\nu_{VENT}(1 - d_{VENT}\delta_{VENT})p_{ifr}VENT + \ldots$$

$$\nu_{VENTC}(1 - d_{VENTC}\delta_{VENTC})p_{ifr}VENTC + \ldots$$

$$\nu_{VENTC}(1 - d_{VENTC}\delta_{VENTC})p_{ifr}ICUCV + \ldots$$

$$\nu_{HC}p_{O2}(1 - \delta_{HCICUO2})p_{ifr}HCICU + \ldots$$

$$\nu_{HC}(1 - p_{O2})(1 - \delta_{HCICU})p_{ifr}HCICU + \ldots$$

$$\nu_{VENTC}(1 - \delta_{HCVENT})p_{ifr}HCV + \ldots$$

$$v_{dr}VR - vAR + 1/v_{iso}Z - q_rR + 1/q_rQR + A_{age}R - \mu R$$

Previously infected individuals, currently quarantining

$$\frac{dQR}{dt} = v_iQI + v_iQC + v_{d_r}QVR + q_rR - 1/v_qQR + A_{age}QR - \mu QR$$

Infected and incubating, from previously vaccinated individuals

$$\frac{dEV}{dt} = (1 - p_V) * \lambda V - \gamma EV - q_rEV + 1/v_qQEV + A_{age}EV - \mu EV$$

Infected and incubating, from previously vaccinated individuals, currently quarantining

$$\frac{dQEV}{dt} = (1 - p_V) * \lambda_qQV - \gamma QEV + q_rEV - 1/v_qQEV + A_{age}QEV - \mu QEV$$

Infected and incubating, from previously infected

$$\frac{dER}{dt} = \lambda\sigma_RR - \gamma ER - q_rER + 1/v_qQER + A_{age}ER - \mu ER$$

Infected and incubating, from previously infected, currently quarantining

$$\frac{dQER}{dt} = \lambda_q\sigma_R \quad QR - \gamma QER + q_rER - 1/v_qQER + A_{age}QER - \mu QER$$

Vaccinated individuals from susceptible

$$\frac{dV}{dt} = vAS - (1 - p_V) * \lambda V + \omega VR - v_d V - q_r V + A_{age} V - \mu V$$

Vaccinated individuals from susceptible, currently quarantining

$$\frac{dQV}{dt} = -(1 - p_V) * \lambda_q QV + \omega QVR + q_r V - 1/v_q QV + A_{age} QV - \mu QV$$

Vaccinated individuals (after being previously infected, recovered)

$$\frac{dVR}{dt} = vAE + vAI + vAR - (1 - p_{VR}) * \lambda VR - v_{d_r} VR - \omega VR - q_r VR + 1/v_q QVR + A_{age} VR - \mu VR$$

Vaccinated individuals (after being previously infected, recovered) (currently quarantining)

$$\frac{dQVR}{dt} = -(1 - p_{VR}) * \lambda QVR - v_{dr} QVR - \omega QVR + q_r VR + A_{age} QVR - \mu QVR$$

Infected and incubating, from previously infected and vaccinated

$$\frac{dEVR}{dt} = (1 - p_{VR}) * \lambda VR - \gamma EVR - q_r EVR + 1/v_q QEVR + A_{age} EVR - \mu EVR$$

Infected and incubating, from previously infected and vaccinated (currently quarantining)

$$\frac{dQEVR}{dt} = (1 - p_{VR}) * \lambda_q QVR - \gamma QEVR + q_r EVR - 1/q_d QEVR + A_{age} QEVR - \mu QEVR$$

States involving hospitalization (or requiring hospitalization)

Hospitalized

$\frac{dH}{dt} = \ldots$

$\gamma p_{ihr}(1 - p_{ICU})(1 - p_{crit}) r_H E \ldots$

$+\gamma p_{ihr}(1 - p_{ICU})(1 - p_{crit}) r_H QE \ldots$

$+\gamma p_{ihr} \sigma_{EV}(1 - p_{ICUV})(1 - p_{critH}) r_H EV \ldots$

$+\gamma p_{ihr} \sigma_{ER}(1 - p_{ICUV})(1 - p_{critH}) r_H QEV \ldots$

$+\gamma p_{ihr} \sigma_{EVR}(1 - p_{ICUVR})(1 - p_{critH}) r_H EVR \ldots$

$$+\gamma p_{ihr}\sigma_{EVR}(1-p_{ICUVR})(1-p_{critH})r_H QEVR\ldots$$

$$+\gamma p_{ihr}\sigma_{ER}(1-p_{ICUR})(1-p_{critH})r_H ER\ldots$$

$$+\gamma p_{ihr}\sigma_{ER}(1-p_{ICUR})(1-p_{critH})r_H QER\ldots$$

$$-\nu_H H + A_{age}H - \mu H$$

Requiring hospitalization, not hospitalized due to capacity

$$\frac{dHC}{dt}=\ldots$$

$$+\gamma p_{ihr}(1-p_{ICU})(1-r_H)E$$

$$+\gamma p_{ihr}(1-p_{ICU})p_{crit}r_H E$$

$$+\gamma p_{ihr}(1-p_{ICU})(1-r_H)QE$$

$$+\gamma p_{ihr}(1-p_{ICU})p_{crit}r_H QE$$

$$+\gamma p_{ihr}\sigma_{EV}(1-p_{ICUV})(1-r_H)EV$$

$$+\gamma p_{ihr}\sigma_{EV}(1-p_{ICUV})p_{crit}r_H EV$$

$$+\gamma p_{ihr}\sigma_{EVR}(1-p_{ICUVR})(1-r_H)EVR\ldots$$

$$+\gamma p_{ihr}\sigma_{EVR}(1-p_{ICUVR})p_{crit}r_H EVR\ldots$$

$$+\gamma p_{ihr}\sigma_{ER}(1-p_{ICUR})(1-r_H)ER\ldots$$

$$+\gamma p_{ihr}\sigma_{ER}(1-p_{ICUR})p_{crit}r_H ER\ldots$$

$$+\gamma p_{ihr}\sigma_{EV}(1-p_{ICUV})(1-r_H)QEV\ldots$$

$$+\gamma p_{ihr}\sigma_{EV}(1-p_{ICUV})p_{crit}r_H QEV\ldots$$

$$+\gamma p_{ihr}\sigma_{EVR}(1-p_{ICUVR})(1-r_H)QEVR\ldots$$

$$+\gamma p_{ihr}\sigma_{EVR}(1-p_{ICUVR})p_{crit}r_H QEVR\ldots$$

$$+\gamma p_{ihr}\sigma_{ER}(1-p_{ICUR})(1-r_H)QER\ldots$$

$$+\gamma p_{ihr}\sigma_{ER}(1-p_{ICUR})p_{crit}r_H QER\ldots$$

$$+u_{HC}A_u HC\ldots$$

$$- \nu_{HC}HC + A_{age}HC - \mu HC$$

Severe infection, hospitalized, but placed in surge ward

$$\frac{dHCV}{dt} = \ldots$$

$$+\gamma(1 - r_{ICU})p_{ihr}p_{ICU}p_{VENTD}E\ldots$$

$$+\ \gamma(1 - r_{ICU})p_{ihr}p_{ICU}p_{VENTD}QE\ldots$$

$$+\gamma(1 - r_{ICU})\sigma_{EV}p_{ihr}p_{ICUV}p_{VENTV}EV\ldots$$

$$+\gamma(1 - r_{ICU})\sigma_{EV}p_{ihr}p_{ICUV}p_{VENTV}QEV\ldots$$

$$+\gamma(1 - r_{ICU})\sigma_{EVR}p_{ihr}p_{ICUVR}p_{VENTVR}EVR\ldots$$

$$+\gamma(1 - r_{ICU})\sigma_{EVR}p_{ihr}p_{ICUVR}p_{VENTVR}QEVR\ldots$$

$$+\gamma(1 - r_{ICU})\sigma_{ER}p_{ihr}p_{ICUR}p_{VENTR}ER\ldots$$

$$+\gamma(1 - r_{ICU})\sigma_{ER}p_{ihr}p_{ICUR}p_{VENTR}QER\ldots$$

$$-u_{HCV}A_u HCV\ldots$$

$$-\nu_{VENTC}HCV + A_{age}HCV - \mu HCV$$

Severe infection, hospitalized, requiring ICU, not but not granted ICU due to capacity

$$\frac{dHCICU}{dt} = \ldots$$

$$+\gamma p_{ihr}p_{ICU}(1 - r_{ICU})(1 - p_{VENTD}\ )E\ldots$$

$$+\gamma p_{ihr}p_{ICU}(1 - r_{ICU})(1 - p_{VENTD})QE\ldots$$

$$+\gamma p_{ihr}\sigma_{EV}p_{ICUV}(1 - r_{ICU})(1 - p_{VENTV})EV\ldots$$

$$+\gamma p_{ihr}\sigma_{EV}p_{ICUV}(1 - r_{ICU})(1 - p_{VENTV})QEV\ldots$$

$$+\gamma p_{ihr}\sigma_{EVR}p_{ICUVR}(1 - r_{ICU})(1 - p_{VENTVR})EVR\ldots$$

$$+\gamma p_{ihr}\sigma_{EVR}p_{ICUVR}(1 - r_{ICU})(1 - p_{VENTVR})QEVR\ldots$$

$$+\gamma p_{ihr}\sigma_{ER}p_{ICUR}(1 - r_{ICU})(1 - p_{VENTR})ER\ldots$$

$$+\gamma p_{ihr}\sigma_{ER}p_{ICUR}(1 - r_{ICU})(1 - p_{VENTR})QER\ldots$$

$$- u_{HCICU} A_u HCICU \ldots$$

$$- \nu_{HC} HCICU + A_{age} HCICU - \mu HCICU$$

Severe infection, requiring ICU

$$\frac{dICU}{dt} = \frac{1}{2}(1 - p_{crit}) ICUC + \ldots$$

$$+\gamma r_{ICU} p_{ihr} p_{ICU} (1 - p_{crit})(1 - p_{VENTD}) E \ldots$$

$$+\gamma r_{ICU} p_{ihr} p_{ICU} (1 - p_{crit})(1 - p_{VENTD}) QE \ldots$$

$$+\gamma r_{ICU} \sigma_{EV} p_{ihr} p_{ICUV} (1 - p_{crit})(1 - p_{VENTV}) EV \ldots$$

$$+\gamma r_{ICU} \sigma_{EV} p_{ihr} p_{ICUV} (1 - p_{crit})(1 - p_{VENTV}) QEV \ldots$$

$$+\gamma r_{ICU} \sigma_{EVR} p_{ihr} p_{ICUVR} (1 - p_{crit})(1 - p_{VENTVR}) EVR \ldots$$

$$+\gamma r_{ICU} \sigma_{EVR} p_{ihr} p_{ICUVR} (1 - p_{crit})(1 - p_{VENTVR}) QEVR \ldots$$

$$+\gamma r_{ICU} \sigma_{ER} p_{ihr} p_{ICUR} (1 - p_{crit})(1 - p_{VENTR}) ER \ldots$$

$$+\gamma r_{ICU} \sigma_{ER} p_{ihr} p_{ICUR} (1 - p_{crit})(1 - p_{VENTR}) QER \ldots$$

$$-\nu_{ICU} ICU + A_{age} ICU - \mu_{ICU}$$

Severe infection, requiring ICU but not receiving ICU due to capacity

$$\frac{dICUC}{dt} = -\frac{1}{2}(1 - p_{crit}) ICUC \ldots$$

$$+\gamma r_{ICU} p_{ihr} p_{ICU} p_{crit} (1 - p_{VENTD}) E \ldots$$

$$+\gamma r_{ICU} p_{ihr} p_{ICU} p_{crit} (1 - p_{VENTD}) QE \ldots$$

$$+\gamma r_{ICU} \sigma_{EV} p_{ihr} p_{ICUV} p_{crit} (1 - p_{VENTV}) EV \ldots$$

$$+\gamma r_{ICU} \sigma_{EV} p_{ihr} p_{ICUV} p_{crit} (1 - p_{VENTV}) QEV \ldots$$

$$+\gamma r_{ICU} \sigma_{EVR} p_{ihr} p_{ICUVR} p_{crit} (1 - p_{VENTVR}) EVR \ldots$$

$$+\gamma r_{ICU} \sigma_{EVR} p_{ihr} p_{ICUVR} p_{crit} (1 - p_{VENTVR}) QEVR \ldots$$

$$+\gamma r_{ICU} \sigma_{ER} p_{ihr} p_{ICUR} p_{crit} (1 - p_{VENTR}) ER \ldots$$

$$+\gamma r_{ICU} \sigma_{ER} p_{ihr} p_{ICUR} p_{crit} (1 - p_{VENTR}) QER \ldots$$

$$-\nu_{ICUC}ICUC + A_{age}ICUC - \mu ICUC$$

Severe infection, hospitalized, requiring ventilator but placed in a surge ward

$$\frac{dICUCV}{dt} = -\frac{1}{2}(1 - p_{critV})ICUCV\ldots$$

$$+\gamma r_{ICU} p_{ihr} p_{ICU} p_{crit}\, p_{VENTD} E\ldots$$

$$+\gamma r_{ICU} \sigma_{EV} p_{ihr} p_{ICUV} p_{crit}\, p_{VENTV} EV\ldots$$

$$+\gamma r_{ICU} \sigma_{EVR} p_{ihr} p_{ICUVR} p_{crit}\, p_{VENTVR} EVR\ldots$$

$$+\gamma r_{ICU} \sigma_{ER} p_{ihr} p_{ICUR} p_{crit}\, p_{VENTR} ER\ldots$$

$$+\gamma r_{ICU} p_{ihr} p_{ICU} p_{crit}\, p_{VENTD} QE\ldots$$

$$+\gamma r_{ICU} \sigma_{EV} p_{ihr} p_{ICUV} p_{crit}\, p_{VENTV} QEV\ldots$$

$$+\gamma r_{ICU} \sigma_{EVR} p_{ihr} p_{ICUVR} p_{crit}\, p_{VENTVR} QEVR\ldots$$

$$+\gamma r_{ICU} \sigma_{ER} p_{ihr} p_{ICUR} p_{crit}\, p_{VENTR} QER\ldots$$

$$-\nu_{VENTC}ICUCV + A_{age}ICUCV - \mu ICUCV$$

Severe infection, hospitalized in ICU on a ventilator

$$\frac{dVENT}{dt} = \ldots$$

$$\frac{1}{2}(1 - p_{critV})VENTC + \frac{1}{2}(1 - p_{critV})ICUCV\ldots$$

$$+\gamma r_{ICU} p_{ihr} p_{ICU}(1 - p_{crit})(1 - p_{critV})\, p_{VENTD} E\ldots$$

$$+\gamma r_{ICU} p_{ihr} p_{ICU}(1 - p_{crit})(1 - p_{critV})\, p_{VENTD} QE\ldots$$

$$+\gamma r_{ICU} \sigma_{EV} p_{ihr} p_{ICUV}(1 - p_{crit})(1 - p_{critV})\, p_{VENTV} EV\ldots$$

$$+\gamma r_{ICU} \sigma_{EV} p_{ihr} p_{ICUV}(1 - p_{crit})(1 - p_{critV})\, p_{VENTV} QEV\ldots$$

$$+\gamma r_{ICU} \sigma_{EVR} p_{ihr} p_{ICUVR}(1 - p_{crit})(1 - p_{critV})\, p_{VENTVR} EVR\ldots$$

$$+\gamma r_{ICU} \sigma_{EVR} p_{ihr} p_{ICUVR}(1 - p_{crit})(1 - p_{critV})\, p_{VENTVR} QEVR\ldots$$

$$+\gamma r_{ICU} \sigma_{ER} p_{ihr} p_{ICUR}(1 - p_{crit})(1 - p_{critV})\, p_{VENTR} ER\ldots$$

$$+\gamma r_{ICU} \sigma_{ER} p_{ihr} p_{ICUR}(1 - p_{crit})(1 - p_{critV})\, p_{VENTR} QER\ldots$$

$$-\nu_{VENT}VENT + A_{age}VENT - \mu VENT$$

Severe infection, hospitalized in ICU requiring a ventilator but not on one due to capacity

$$\frac{dVENTC}{dt} = \ldots$$

$$-\frac{1}{2}(1 - p_{critV})VENTC\ldots$$

$$+\gamma r_{ICU} p_{ihr} p_{ICU}(1 - p_{crit})p_{critV}\ p_{VENTD}E\ldots$$

$$+\gamma r_{ICU} p_{ihr} p_{ICU}(1 - p_{crit})p_{critV}\ p_{VENTD}QE\ldots$$

$$+\gamma r_{ICU}\sigma_{EV} p_{ihr} p_{ICUV}(1 - p_{crit})p_{critV}\ p_{VENTV}EV\ldots$$

$$+\gamma r_{ICU}\sigma_{EV} p_{ihr} p_{ICUV}(1 - p_{crit})p_{critV}\ p_{VENTV}QEV\ldots$$

$$+\gamma r_{ICU}\sigma_{EVR} p_{ihr} p_{ICUVR}(1 - p_{crit})p_{critV}\ p_{VENTVR}EVR\ldots$$

$$+\gamma r_{ICU}\sigma_{EVR} p_{ihr} p_{ICUVR}(1 - p_{crit})p_{critV}\ p_{VENTVR}QEVR\ldots$$

$$+\gamma r_{ICU}\sigma_{ER} p_{ihr} p_{ICUR}(1 - p_{crit})p_{critV}\ p_{VENTR}ER\ldots$$

$$+\gamma r_{ICU}\sigma_{ER} p_{ihr} p_{ICUR}(1 - p_{crit})p_{critV}\ p_{VENTR}QER\ldots$$

$$-\nu_{VENTC}VENTC + A_{age}VENTC - \mu_{VENTC}$$

Force of infection **(**lambda, lam, λ**)**

$$\lambda = \left(1 - max(hand, mask)\right) p.s.W$$

$$\frac{\rho E + I + CL + m + (1 - s_{iso}\ \ )(X + HC + HCICU + HCV) + \rho_s(H + ICU + VENT + ICUC + ICUCV + VENTC)}{P} \ldots$$

$$+(1 - max(hand, mask))p.s.(1 - q_{other})W_{other}\frac{\rho QE + QI + QC + QEV + QEVR + QER)}{P}$$

where

$$mask = maskeff * maskcov;\ hand\ =\ handeff * handcov$$

For those under quarantine, this is adjusted in the following manner:

$$\lambda_q = \left(1 - max(hand, mask)\right) ps(1 - q_{home})W_{home}$$

$$\frac{(1 - s_{iso}\ \ )(X + HHC + \rho QE + QI + QC + QEV + QEVR + QER)}{P} \ldots$$

$$+\left(1 - max(hand, mask)\right) ps(1 - q_{other})W_{other}$$

$$\frac{\rho E + I + CL + m + (1 - s_{iso}\ \ )(X + HC + HCICU + HCV) + \rho_s(H + ICU + VENT + ICUC + ICUCV + VENTC)}{P}$$

where

$$P = S + E + I + R + X + Z + CL \ldots$$

$$+V + EV + ER + EVR + VR \ldots$$

$$+H + HC + ICU + ICUC + ICUCV + Vent + VentC + HCICU + HCV \ldots$$

$$+QS + QE + QI + QR + QC + QEV + QV + QER + QEVR + QVR$$

Quarantine rate **(q_r)**

Quarantine rate (for all non-hospitalized compartments that transition into a quarantine state) is calculated in the following manner (see here):

$$q_{r\,=}\, r_q(1 + exp(-10(\sigma_q/2 \; - \; Q))$$

Proportion of the population in quarantine:

$$Q \;= (\; \Sigma QS + \Sigma QE + \Sigma QI + \Sigma QC + \Sigma QR + \Sigma QV + \Sigma QEV + \Sigma QEVR + \Sigma QER + \Sigma QVR)/(\Sigma P)$$

Vaccination rate (v)

Vaccination rate is calculated in the following manner (see here):

$$v = -log(1 - v_{cov})/v_c$$

Model Parameterization

Several pieces of data were used to parameterize the model for simulations of the COVID-19 outbreak in Jordan. Parameters governing population size (of ≈100 million individuals), demographic age-structure, fertility, and natural mortality were taken from the 2019 UN Revision of World Population Prospects. Contact data were informed using Prem et al. (*2*) and average household size is 4.7 (Jordan MoH). Parameters governing infection and disease progression follow the literature on SARS-CoV-2 and COVID-19 and are provided in detail in the supplemental data. Parameters governing healthcare capacity were determined via discussions with in-country partners. Jordan country partners specified in the model that all ICUs are with ventilators with the assumption that sever, and critical patients are admitted to the ICU when requiring mechanical ventilation. Other patients receive care in normal hospital beds with or without oxygen supply. We assumed that the maximum number of hospital beds (3894), the maximum number of ICU beds with ventilators (834) during this period. Parameters governing the time course of hospitalized infections were determined via discussions with in-country partners and are specific to Jordan. Because of various travel restrictions imposed by the Jordanian government in 2020, we assumed that there were no imported cases over the modeled period. Waning immunity was not included in this model.

Within the CoMo model framework, model calibration was performed by adjusting the probability of transmission given an infectious contact and the date of introduction of SARS-CoV-2 into the country. These calibrations were performed visually using the CoMo model application. We were justified in performing visual calibrations given the interactive nature of the user platform, the short time frame in which results were required by the Jordanian government, and a focus on the relative impact of different interventions on future transmission rather than absolute numbers of cases or deaths.

Using the calibrated model, we simulated several different scenarios of the implementation of PHSM for Jordan in 2020 from end of October 2020 until end of January 2021. Parameters governing the PHSM scenarios were set according to discussions with the Jordan Ministry of Health as part of our participatory modeling process. We set parameter values to simulate the changing of PHSM in Jordan at various time points throughout 2020.

Model Inputs

Appendix Tables 3–11 describe the model inputs.

References

1. Aguas R, White L, Hupert N, Shretta R, Pan-Ngum W, Celhay O, et al.; CoMo Consortium. Modelling the COVID-19 pandemic in context: an international participatory approach. BMJ Glob Health. 2020;5:e003126. PubMed https://doi.org/10.1136/bmjgh-2020-003126

2. Prem K, Cook AR, Jit M. Projecting social contact matrices in 152 countries using contact surveys and demographic data. PLOS Comput Biol. 2017;13:e1005697. PubMed https://doi.org/10.1371/journal.pcbi.1005697

Appendix Table 1. Variables. There are 31 different variables that make up the population, 35 derivatives returned from the solver (4 for convenience).

Symbol	Definition
S	Susceptible
E	Infected and incubating
I	Infectious and asymptomatic following incubation
R	Recovered and immune
X	Self-isolating
H	Severe infection: hospitalized (Aguas et al., 2020)
HC	Severe infection, requiring hospitalization, not hospitalized due to lack of capacity (Aguas et al., 2020)
HCICU	Severe infection, hospitalized, requiring ICU, not but not granted ICU due to capacity
HCV	Severe infection, hospitalized, but placed in surge ward
ICU	Severe infection, requiring ICU
ICUC	Severe infection, requiring ICU, not hospitalized due to capacity
ICUCV	Severe infection, hospitalized, requiring ventilator but placed in surge ward
Vent	Severe infection, hospitalized in ICU on a ventilator
VentC	Severe infection, hospitalized in ICU requiring a ventilator but not on one
C	Infectious and mildly symptomatic following incubation (not in the total popn count, L416 comoOde.cpp)
CM	(not in the total popn count, L416 comoOde.cpp)
V	Vaccinated (from susceptible)
QS	Susceptible, quarantining
QE	Infected and incubating, quarantining
QI	Infectious and asymptomatic following incubation, quarantining
QR	Recovered and immune, quarantining
CL	Total cases?
Z	Quarantined due to testing
EV	Infected and incubating, from previously vaccinated individuals
ER	Infected and incubating, from previously infected individuals
EVR	Vaccinated (from previously infected), exposed (from ineffective vaccine).
VR	Vaccinated (from previously infected)
QV	Vaccinated (from susceptible), quarantining
QEV	Vaccinated (from susceptible), exposed (from ineffective vaccine), quarantining
QEVR	Previously infected, exposed (from ineffective vaccine), quarantining
QER	Exposed, quarantining, previously infected
QVR	Vaccinated, quarantining, previously infected
Ab	Those with antibody response. Not part of the population, simply calculated in the solver.

Appendix Table 2. Parameters

Parameter name (as used in code)	Symbol	Description	Units
Severity-Mortality Parameters			
ifr?	p_{ifr}	Age-based relative fatality rate in well-resourced scenario	%
ihr_col2	p_{ihr}	Probability of an infection being severe (requiring hospitalization) by age	%
Population parameters			
mu	μ	1/age-dependent non-Covid-19-related death rate	days
B	b	1/ age-dependent fertility rate	days
General parameters			
date_range_simul_start	t_0	Date range of simulation – START	
date_range_simul_end	t_T	Date range of simulation – END	
init	–	Number of exposed people at start date	
pre	–	Proportion of population with partial immunity at the start date	
p	p_E	Probability of infection given contact	
report	p_{ar}	Percentage of all asymptomatic infections that are reported	%
reportc	p_{cr}	Percentage of all symptomatic infections that are reported	%
reporth_g	p_{gr}	Percentage of denied hospitalizations that are reported	%
reporth	r_H	Percentage of non-severe hospitalizations that are appropriately treated (realized)	%
reporth_ICU	r_{ICU}	Percentage of severe hospitalizations that are appropriately treated (realized)	%
report_v	p_{avr}	Percentage of all asymptomatic infections in previously vaccinated people that are reported	
report_vr	p_{avrr}	Percentage of all asymptomatic infections in previously vaccinated and exposed people that are reported	
report_r	p_{arr}	Percentage of all asymptomatic infections in previously infected people that are reported	

Parameter name (as used in code)	Symbol	Description	Units
report_cv	p_{cvr}	Percentage of all symptomatic infections in previously vaccinated people that are reported	
report_cvr	p_{cvrr}	Percentage of all symptomatic infections in previously vaccinated and exposed people that are reported	
report_cr	p_{crr}	Percentage of all symptomatic infections in previously infected people that are reported	
iterations	–	Iterations (1 to 10,000)	
noise	–	Noise (0.01 to 0.2)	
confidence	–	Confidence (5 to 25)	
sample_size	–	Average sample size for seroprevalence	
Country Area parameters			
country_contact	C	Country, used for social Contacts Data	
household_size	h	Mean Household size	individuals
mean_imports	m	Mean number of infectious migrants per day; note that the code includes a 'travel ban efficacy' parameter but assumed set to zero (see here).	individuals
Virus parameters			
rho	ρ	relative infectiousness of incubation phase	
gamma	γ	incubation period	Days
nui	ν_i	Average duration of symptomatic infection period	Days
phi	ϕ	Month of peak infectivity of the virus (1, 2, …, 12)	
amp	a	Annual variation in infectivity of the virus	
omega	ω	duration of naturally acquired immunity	Years
pclin	p_{clin}	Probability upon infection of developing clinical symptoms	
prob_icu	p_{ICU}	Probability upon hospitalization of requiring ICU admission	%
prob_vent	p_{VENT}	Probability upon admission to the ICU of requiring a ventilator	%
propo2	p_{O2}	Proportion of hospitalized patients needing O2	
pclin_v	p_{clinV}	Probability upon infection of developing clinical symptoms if previously vaccinated	
pclin_vr	p_{clinVR}	Probability upon infection of developing clinical symptoms if previously vaccinated and exposed	
pclin_r	p_{clinR}	Probability upon infection of developing clinical symptoms if previously infected	
prob_icu_v	p_{ICUV}	Probability upon hospitalization of requiring ICU admission if previously vaccinated	%
prob_icu_vr	p_{ICUVR}	Probability upon hospitalization of requiring ICU admission if previously vaccinated and exposed	%
prob_icu_r	p_{ICUR}	Probability upon hospitalization of requiring ICU admission if previously infected	%
prob_v	p_{VENTD}	Derived. Probability upon admission to the ICU of requiring a ventilator. Essentially the same as prob_vent, if dexamethsone is being used then this param is prob_vent*vent_dex, else it is prob_vent (see here).	
prob_v_v	p_{VENTV}	Probability upon admission to the ICU of requiring a ventilator if previously vaccinated	%
prob_v_vr	p_{VENTVR}	Probability upon admission to the ICU of requiring a ventilator if previously vaccinated and exposed	%
prob_v_r	p_{VENTR}	Probability upon admission to the ICU of requiring a ventilator if previously infected	%
sigmaR	σ_R	Probability of infection of people that have recovered from a previous infection	
sigmaEV	σ_{EV}	Probability of requiring hospitalization if previously vaccinated	
sigmaER	σ_{ER}	Probability of requiring hospitalization if previously infected	
sigmaEVR	σ_{EVR}	Probability of requiring hospitalization if previously infected and vaccinated	
seroneg	$seroneg$	Days from seropositve to seronegative	Days
Hospitalization parameters			
beds_available	–	Maximum number of hospital surge beds	beds
icu_beds_available	–	Maximum number of ICU beds without ventilators	beds
ventilators_available	–	Maximum number of ICU beds with ventilators	beds
rhos	ρ_s	Relative percentage of regular daily contacts when hospitalized	
ihr_scaling	–	Scaling factor for infection hospitalization rate: (0.1 to 5)	
pdeath_h	δ_H	Probability of dying when hospitalized (not requiring O2)	
pdeath_ho	δ_{HO2}	Probability of dying when hospitalized if requiring O2	
pdeath_hc	δ_{HC}	Probability of dying when denied hospitalization (not requiring O2)	
pdeath_hco	δ_{HCO2}	Probability of dying when denied hospitalization if requiring O2	
pdeath_icu	δ_{ICU}	Probability of dying when admitted to ICU (not requiring O2)	

Parameter name (as used in code)	Symbol	Description	Units
pdeath_icuo	δ_{ICUO2}	Probability of dying when admitted to ICU if requiring O2	
pdeath_icuc	δ_{ICUC}	Probability of dying when admission to ICU denied (not requiring O2)	
pdeath_icuco	δ_{ICUCO2}	Probability of dying when admission to ICU denied if requiring O2	
pdeath_vent	δ_{VENT}	Probability of dying when ventilated	
pdeath_ventc	δ_{VENTC}	Probability of dying when ventilator denied	
pdeath_vent_hc	δ_{HCVENT}	Probability of dying when ventilator required and not going to hospital	
pdeath_icu_hc	δ_{HCICU}	Probability of dying when ICU required (not O2) and not going to hospital	
pdeath_icu_hco	$\delta_{HCICUO2}$	Probability of dying when ICU required (requiring O2) and not going to hospital	
nus	ν_H	Average duration of hospitalized infection	Days
nusc	ν_{HC}	Average duration of infection requiring hospitalization but not receiving it due to capacity (assumed same as nus parameter).	Days
nu_icu	ν_{ICU}	Average duration of ICU infection	Days
nu_icuc	ν_{ICUC}	Average duration of infection of individual requiring ICU infection but not receiving it due to capacity (assumed same as nu_icu parameter).	Days
nu_vent	ν_{VENT}	Average duration of ventilated infected.	Days
nu_ventc	ν_{VENTC}	Average duration of infection requiring ventilation but not receiving it due to capacity (assumed same as nu_vent parameter).	Days
Interventions Parameters			
selfis_eff	s_{iso}	Adherence to self-isolation	%
screen_overdispersion	–	Overdispersion: (1, 2, 3, 4 or 5)	
screen_test_sens	s_{screen}	Test sensitivity	%
(*Self-isolation) Household Isolation Parameters			
quarantine_days	v_q	Days in isolation for average person	Days
quarantine_effort	e_q	Days to implement maximum quarantine coverage (1 to 5)	days
quarantine_eff_other	q_{other}	Decrease in the number of other contacts when quarantined	%
quarantine_eff_home	q_{home}	Increase in the number of contacts at home when quarantined	%
Social Distancing Parameters			
dist_eff	$disteff$	Adherence to social distancing	%
Handwashing Parameters			
hand_eff	$handeff$	Efficacy of handwashing (0%–25%); note that only the maximum of handwashing or mask-wearing (efficacy * coverage) is used in transmission calculations. Coverage of handwashing (proportion of individuals following handwashing guidelines) is denoted with $handcov$.	%
Mask-wearing Parameters			
mask_eff	$maskeff$	Efficacy of mask wearing (0%–35%); note that only the maximum of handwashing or mask-wearing (efficacy * coverage) is used in transmission calculations. Coverage of mask wearing (proportion of population following mask wearing guidelines) is denoted with $maskcov$.	%
Working at Home Parameters			
work_eff	$workeff$	Efficacy of working from home	%
w2h	–	Home contacts inflation due to working from home:	%
School Closures Parameters			
s2h	–	Home contacts inflation due to school closure	%
Shielding the Elderly Parameters			
cocoon_eff	–	Efficacy of shielding the elderly	%
age_cocoon	–	Minimum age for elderly shielding (0 to 100)	y.o.
Vaccination Parameters			
vac_campaign	v_c	Time to reach target coverage in vaccination campaign (1 to 52)	weeks
vac_dur	v_d	1 / duration of vaccine efficacious period	Years
vac_dur_r	v_{dr}	1 / duration of vaccine efficacious period if previously infected	Years
vaccine_eff	p_V	Vaccine efficacy	
vaccine_eff_r	p_{VR}	Vaccine efficacy if previously infected	
Mass Testing Parameters			
mass_test_sens	s_{mass}	Sensitivity of mass testing	%
isolation_days	v_{iso}	Isolation days	days
Dexamethasone Parameters			
dexo2	d_{O2}	Relative risk of dying if needing O2 and taking dexamethasone	%
dexv	d_{VENT}	Relative risk of dying if needing ventilation and taking dexamethasone	%
dexo2c	d_{O2C}	Relative risk of dying if needing but not receiving O2 and taking dexamethasone	%
dexvc	d_{VENTC}	Relative risk of dying if needing but not receiving ventilation and taking dexamethasone	%

Parameter name (as used in code)	Symbol	Description	Units
vent_dex	–	Change in ventilation requirement if given dexamethasone (multiplier on prob_vent). See description of prob_v parameter.	%
Derived parameters			
aging	A_{age}	Aging matrix (assuming states are vectors with elements for each age).	
quarantine_rate	q_r	Quarantine rate (see below)	
vaccinate	v	Vaccination rate	
age_vaccine_vector	A_v	Vector of vaccination uptake by age	
lam	λ	Force of infection including impact of PHSM (lambda)	
lamq	λ_q	Force of infection including impact of PHSM (lambda) for those under quarantine	
critH	p_{critH}	Calculated from splines.	
crit	p_{crit}		
critV	p_{critV}		
age_testing_vector	A_u	Vector of test rates by age	
ratetestE	u_E	Testing rate of the E class	
ratetestEV	u_{EV}	Testing rate of the EV class	
ratetestEVR	u_{EVR}	Testing rate of the EVR class	
ratetestER	u_{ER}	Testing rate of the ER class	
ratetestI	u_I	Testing rate of the I class	
ratetestC	u_{CL}	Testing rate of the CL class	
ratetestHC	u_{HC}	Testing rate of the HC class	
ratetestHCICU	u_{HCICU}	Testing rate of the HCICU class	
ratetestHCV	u_{HCV}	Testing rate of the HCV class	
screen_eff	s_{screen}	Efficacy of screening	
seas	s	Seasonality factor; $s = 1 + a\,cos\,(2\pi(t - (365.25\phi/12))/365.25)$	
P	P	Total population	
selfis_cov	c_{iso}	Coverage of self-isolation	
selfis (here)			

Appendix Table 3. Cases

Inputs	Value(s)	Input Source
Number of reported COVID19 cases per day	Cases reported between 24/02/2020 and 15/10/2020	Jordan MoH
Daily COVID19 deaths	deaths reported between 14/02/2020 and 15/10/2020	Jordan MoH

Appendix Table 4. Severity and Mortality

Age category	Age-based relative fatality rate in well-resourced scenario (%) data source: https://science.sciencemag.org/content/sci/suppl/2020/05/12/science.abc3517.DC1/abc3517_Salje_SM_rev2.pdf	Age-stratum-specific hospitalization (proportion of all (asymptomatic + symptomatic) infections that lead to hospitalization) (%)
0–5 y.o.	0.6	0.1
5–10 y.o.	0.6	0.1
10–15 y.o.	0.6	0.1
15–20 y.o.	0.6	0.1
20–25 y.o.	1.1	0.5
25–30 y.o.	1.1	0.5
30–35 y.o.	1.9	1.1
35–40 y.o.	1.9	1.1
40–45 y.o.	3.3	1.4
45–50 y.o.	3.3	1.4
50–55 y.o.	6.5	2.9
55–60 y.o.	6.5	2.9
60–65 y.o.	12.6	5.8
65–70 y.o.	12.6	5.8
70–75 y.o.	21	9.3
75–80 y.o.	21	9.3
80–85 y.o.	31.6	26.2
85–90 y.o.	31.6	26.2
90–95 y.o.	31.6	26.2
95–100 y.o.	31.6	26.2
100+ y.o.	31.6	26.2

Appendix Table 5. Population

Age category	Population Data Source: UN 2019 Revision of World Population Prospects.	Number of births per person (ie 0.5* births per woman) per day Data Source: UN 2019 Revision of World Population Prospects[7.]	Deaths per person per day Data Source: UN 2019 Revision of World Population Prospects[7.]
0–4 y.o.	1058122	0	9.5131E-06
5–9 y.o.	1154441	0	8.927E-07
10–14 y.o.	1139559	0	7.212E-07
15–19 y.o.	1040294	3.33108E-05	1.3306E-06
20–24 y.o.	940151	0.000141126	1.8731E-06
25–29 y.o.	862385	0.000206421	2.0039E-06
30–34 y.o.	777482	0.000183412	2.3882E-06
35–39 y.o.	713126	0.000110293	3.1029E-06
40–44 y.o.	606717	3.27423E-05	4.7969E-06
45–49 y.o.	534628	2.2533E-06	7.8137E-06
50–54 y.o.	433223	0	1.23955E-05
55–59 y.o.	322368	0	1.99431E-05
60–64 y.o.	217239	0	3.30424E-05
65–69 y.o.	149039	0	5.96328E-05
70–74 y.o.	111528	0	0.000103811
75–79 y.o.	79223	0	0.000169096
80–84 y.o.	43119	0	0.000280179
85–89 y.o.	16283	0	0.000437
90–94 y.o.	3741	0	0.000637149
95–99 y.o.	444	0	0.001002645
100+ y.o.	28	0	0.015899091

Appendix Table 6. Country Area Parameters

Inputs	Value(s)	Input Source
Social Contacts Data:	Jordan	Prem, K., A.R. Cook, and M. Jit, *Projecting social contact matrices in 152 countries using contact surveys and demographic data.* PLoS Comput Biol, 2017. 13(9): p. e1005697.
Mean Household size:	4.7 individuals	Jordan MoH
Mean number of infectious migrants per day:	0 individuals	As the travel restrictions were implemented early in the pandemic it was assumed to be 0 if effective isolation of infectious migrants were in place.

Appendix Table 7. Calibration Parameters

Parameter	Scenario: 10% of the population infected	Source
Date range of simulation - START	1/3/2020	fitted
Date range of simulation - END	31/1/2021	assumed
Number of exposed people at start date	1	fitted
Probability of infection given contact (0 to 0.2)	0.029	fitted

Appendix Table 8. Virus Parameters

Relative infectiousness of incubation phase:	10%	Assumed value
Average incubation period:	3.5 d	Linton, N.M., et al., *Incubation Period and Other Epidemiologic Characteristics of 2019 Novel Coronavirus Infections with Right Truncation: A Statistical Analysis of Publicly Available Case Data.* J Clin Med, 2020. 9(2).<br>Khalili, M., et al., *Epidemiologic Characteristics of COVID-19; a Systematic Review and Meta-Analysis.* Plos One, 2020. Submitted.<br>Bi, Q., et al Epidemiology *and Transmission of COVID-19 in Shenzhen China: Analysis of 391 cases and 1,286 of their close contacts.* medRxiv, 2020: p. 2020.03.03.20028423.<br>*Coronavirus disease 2019 (COVID-19) pandemic: increased transmission in the EU/EEA and the UK – seventh update*. Available from: https://www.ecdc.europa.eu/sites/default/files/documents/RRA-seventh-update-Outbreak-of-coronavirus-disease-COVID-19.pdf.
Average duration of symptomatic infection period:	4.5 d	Linton, N.M., et al., *Incubation Period and Other Epidemiologic Characteristics of 2019 Novel Coronavirus Infections with Right Truncation: A Statistical Analysis of Publicly Available Case Data.* J Clin Med, 2020. 9(2).
Probability upon infection of developing clinical symptoms:	55%	Mizumoto, K., et al., *Estimating the Asymptomatic Proportion of 2019 Novel Coronavirus onboard the Princess Cruises Ship, 2020.* medRxiv, 2020: p. 2020.02.20.20025866.<br>Day, M., *Covid-19: four fifths of cases are asymptomatic, China figures indicate.* BMJ, 2020. 369: p. m1375.<br>*Coronavirus disease 2019 (COVID-19) pandemic: increased transmission in the EU/EEA and the UK – seventh update*. Available from: https://www.ecdc.europa.eu/sites/default/files/documents/RRA-seventh-update-Outbreak-of-coronavirus-disease-COVID-19.pdf.
Probability upon hospitalization of requiring ICU admission with mechanical ventilation:	10%	Jordan MoH

Appendix Table 9. Hospitalization Parameters

Inputs	Value(s)	Input Source
Maximum number of hospital beds	3894 beds	Jordan MoH
Maximum number of ICU beds with ventilators	834 ventilators	Jordan MoH
Relative percentage of regular daily contacts when hospitalized:	15%	Assumed value
Probability of dying when hospitalized:	35%	https://www1.nyc.gov/site/doh/covid/covid-19-data.page.<br>Zhou, F., et al., *Clinical course and risk factors for mortality of adult inpatients with COVID-19 in Wuhan, China: a retrospective cohort study.* Lancet, 2020. 395(10229): p. 1054–1062.
Probability of dying when denied hospitalization:	50%	Petrilli, C.M., et al., *Factors associated with hospitalization and critical illness among 4,103 patients with COVID-19 disease in New York City.* medRxiv, 2020: p. 2020.04.08.20057794.
Probability of dying when admitted to ICU:	68%	value was recalculated from:<br>Petrilli, C.M., et al., *Factors associated with hospitalization and critical illness among 4,103 patients with COVID-19 disease in New York City.* medRxiv, 2020: p. 2020.04.08.20057794.<br>Lewnard, J.A., et al., *Incidence, clinical outcomes, and transmission dynamics of hospitalized 2019 coronavirus disease among 9,596,321 individuals residing in California and Washington, United States: a prospective cohort study.* medRxiv, 2020: p. 2020.04.12.20062943.<br>To reflect the hospital system in Jordan

Inputs	Value(s)	Input Source
Probability of dying when admission to ICU denied:	85%	value was recalculated from: Petrilli, C.M., et al., *Factors associated with hospitalization and critical illness among 4,103 patients with COVID-19 disease in New York City*. medRxiv, 2020: p. 2020.04.08.20057794. To reflect the hospital system in Jordan
Duration of hospitalized infection:	7 d	Jordan MoH
Duration of ICU with ventilation infection:	14 d	Jordan MoH

Appendix Table 10. Public health and social measures parameters

Self-isolation if Symptomatic	Parameter	Value	Source
	Adherence:	65%	Jordan MoH
(*Self-isolation) Screening			Jordan MoH
	Overdispersion: (1, 2, 3, 4 or 5)	4	Jordan MoH
	Test Sensitivity:	80%	Jordan MoH
(*Self-isolation) Household Isolation			
	Days in isolation for average person:	14 d	Jordan MoH
	Days to implement maximum quarantine coverage: (1 to 5)	2 d	Jordan MoH
	Decrease in the number of other contacts when quarantined:	20%	Jordan MoH
	Increase in the number of contacts at home when quarantined:	100%	Jordan MoH
Social Distancing			
	Adherence:	Varied %	Jordan MoH
Handwashing			
	Efficacy: (0%–25%)	5%	Jordan MoH
Mask Wearing			
	Efficacy: (0%–35%)	35%	Jordan MoH
Working at Home			
	Efficacy:	95%	Jordan MoH
	Home contacts inflation due to working from home:	10%	Jordan MoH
School Closures			
	Home contacts inflation due to school closure:	10%	Jordan MoH

Appendix Table 11. Interventions

Intervention	Date Start	Date End	Value	Unit	Source
Self-isolation if Symptomatic	3/16/2020	9/20/2020	85	%	Jordan MoH
Self-isolation if Symptomatic	9/21/2020	1/31/2021	50	%	Jordan MoH
Social Distancing	3/15/2020	5/1/2020	70	%	Jordan MoH
Social Distancing	5/2/2020	6/1/2020	45	%	Jordan MoH
Social Distancing	6/2/2020	7/1/2020	15	%	Jordan MoH
Social Distancing	7/2/2020	9/15/2020	10	%	Jordan MoH
Social Distancing	9/16/2020	9/25/2020	15	%	Jordan MoH
Social Distancing	9/26/2020	10/29/2020	35	%	Jordan MoH
Social Distancing	10/30/2020	10/31/2020	80	%	Jordan MoH
Social Distancing	11/1/2020	11/5/2020	50	%	Jordan MoH
Social Distancing	11/6/2020	11/7/2020	80	%	Jordan MoH
Social Distancing	11/8/2020	11/12/2020	50	%	Jordan MoH
Social Distancing	11/13/2020	11/14/2020	80	%	Jordan MoH
Social Distancing	11/15/2020	11/19/2020	50	%	Jordan MoH
Social Distancing	11/20/2020	11/21/2020	80	%	Jordan MoH
Social Distancing	11/22/2020	11/26/2020	50	%	Jordan MoH
Social Distancing	11/27/2020	11/28/2020	80	%	Jordan MoH
Social Distancing	11/29/2020	12/3/2020	50	%	Jordan MoH
Social Distancing	12/4/2020	12/5/2020	80	%	Jordan MoH
Social Distancing	12/6/2020	12/10/2020	50	%	Jordan MoH
Social Distancing	12/11/2020	12/12/2020	80	%	Jordan MoH
Social Distancing	12/13/2020	12/17/2020	50	%	Jordan MoH
Social Distancing	12/18/2020	12/19/2020	80	%	Jordan MoH
Social Distancing	12/20/2020	12/24/2020	50	%	Jordan MoH
Social Distancing	12/25/2020	12/26/2020	80	%	Jordan MoH
Social Distancing	12/27/2020	12/31/2020	50	%	Jordan MoH
Social Distancing	1/1/2021	1/2/2021	80	%	Jordan MoH
Social Distancing	1/3/2021	1/7/2021	50	%	Jordan MoH
Social Distancing	1/8/2021	1/9/2021	80	%	Jordan MoH
Social Distancing	1/10/2021	1/14/2021	50	%	Jordan MoH
Social Distancing	1/15/2021	1/16/2021	80	%	Jordan MoH
Social Distancing	1/17/2021	1/21/2021	50	%	Jordan MoH
Social Distancing	1/22/2021	1/23/2021	80	%	Jordan MoH
Social Distancing	1/24/2021	1/28/2021	50	%	Jordan MoH
Social Distancing	1/29/2021	1/30/2021	80	%	Jordan MoH
Social Distancing	1/31/2021	2/4/2021	50	%	Jordan MoH
Social Distancing	2/5/2021	2/6/2021	80	%	Jordan MoH
Social Distancing	2/7/2021	2/11/2021	50	%	Jordan MoH
Social Distancing	2/12/2021	2/13/2021	80	%	Jordan MoH
Social Distancing	2/14/2021	1/31/2021	50	%	Jordan MoH
Handwashing	2/25/2020	2/15/2021	100	%	Jordan MoH
Working at Home	3/17/2020	5/1/2020	70	%	Jordan MoH
Working at Home	5/2/2020	6/1/2020	45	%	Jordan MoH
Working at Home	6/2/2020	9/25/2020	15	%	Jordan MoH
Working at Home	9/26/2020	1/31/2021	35	%	Jordan MoH
School Closures	3/14/2020	9/1/2020	100	%	Jordan MoH
School Closures	9/15/2020	10/5/2020	70	%	Jordan MoH
School Closures	10/6/2020	1/31/2021	100	%	Jordan MoH
